# The Solvation of Ca^2+^ with Gas Phase Clusters of Alcohol Molecules

**DOI:** 10.1007/s13361-019-02263-x

**Published:** 2019-07-08

**Authors:** Khadar Duale, Anthony J. Stace

**Affiliations:** 1 School of Chemistry, The University of Nottingham, University Park, Nottingham, NG7, 2RD UK; 2 School of Biology, Chemistry and Forensic Science, Faculty of Science and Engineering, University of Wolverhampton, Wolverhampton, WV1 1SB UK

**Keywords:** Calcium dication, Clusters, Alcohol molecules, Charge separation, Coulomb fission, Switching reactions

## Abstract

A comprehensive examination of how the identity of an alcohol molecule can change the behavior of a solvated, alkaline earth dication has been undertaken. The metal dication of Ca^2+^ has been clustered with a range of different alcohols to form [Ca(ROH)_*n*_]^2+^ complexes, where *n* lies in the range 2–20. Following collisional activation via electron capture from nitrogen gas, complexes for *n* in the range 2–6 exhibit a switch in reaction product as a function of *n*. For low values, solvated CaOH^+^ is the dominant fragment, but as *n* increases beyond 4, this is displaced by the appearance of solvated CaOR^+^. A separate study of unimolecular metastable decay by [Ca(ROH)_*n*_]^2+^ complexes found evidence of charge separation to form CaOH^+^(ROH)_*n*−1_ + R^+^. For two isomers of butanol, the *n* = 3 complexes were found to follow parallel, but different metastable pathways: one leading to the appearance of CaOH^+^ and another that resulted in proton abstraction to form ROH_2_^+^. These differences have been attributed to the precursor complexes adopting geometries where one ROH molecule occupies a secondary solvation shell. Comparisons were made with a previous study of magnesium complexes; [Mg(ROH)_*n*_]^2+^ show that the difference in second ionization energy Mg^+^ (15.09 eV) as opposed to Ca^+^ (11.88 eV) influences behavior. A complex between Ca^2+^ and 1-chloroethanol is shown to favor the formation of CaCl^+^ as opposed to CaOH^+^ as a unimolecular charge separation product, which is attributed to differences in bond energy in the precursor molecule.

**Electronic supplementary material:**

The online version of this article (10.1007/s13361-019-02263-x) contains supplementary material, which is available to authorized users.

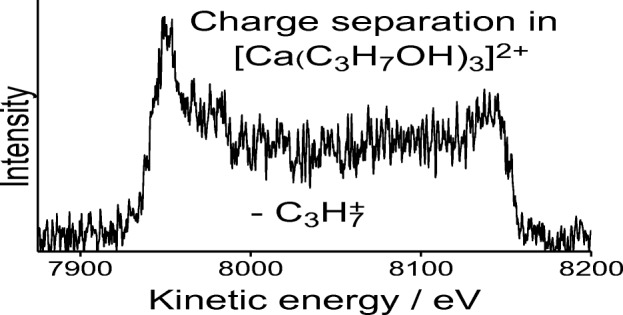

## Introduction

Advances in the techniques available for generating metal dication complexes in the gas phase have made it possible to study a wide range of their chemical and spectroscopic properties [1–3]. Together, the two principal experimental methods for preparing such complexes, namely, electrospray [2] and pick-up [1], have investigated an extensive array of ligands in association with a wide range of metal dications. In this context, the pick-up technique has proved to be particularly versatile, having been shown to generate complexes with ligands ranging from argon [4] through to phosphorus-containing compounds [5]. Limitations in the range of solvents available to electrospray mean that, using this approach, experiments have been primarily restricted to metal dications in association with molecules suspended in water [2]. To date, there has been just one experimental study of the behavior of metal dications in association with ligands that constitute a homologous series [6]. In principle, such a series should provide an opportunity to explore changes in metal ion stability and solvation that may occur as a result of a systematic variation in one or more molecular properties. Such variations could include size (steric effects and differences in geometry between isomers), polarizability, and dipole moment. The earlier study showed the presence of a link between the propensity for metastable decay and the ionization energy of R, the radical that forms the complementary ion as a result of unimolecular charge separation [6]. Presented here are results from a series of experiments that have investigated the behavior of dication complexes consisting of Ca^2+^ in association with clusters composed of methanol, ethanol, 1,2-propanol, 1,2-butanol, t-butanol, and 1-chloroethanol. Observed chemical processes include unimolecular charge separation and the formation of CaOH^+^ and R^+^ and loss of the radicals H⋅ and R⋅, promoted by electron capture. Although there are common patterns of behavior within the range of molecules studied, there are also notable variations that appear to arise from differences in geometry.

The vast majority of previous gas phase studies of Ca^2+^ complexes have been in association with water molecules and the determination of hydration numbers [7–17], water ligand–binding energies, and proton transfer reactions. Kebarle and co-workers [7–10] were able to determine hydration energies by establishing equilibrium conditions in the gas phase. Also observed were charge transfer products resulting from the interaction of doubly charged calcium with water molecules, where the reaction was interpreted in the form of a proton transfer step:

1 $$ {\left[\mathrm{Ca}{\left({\mathrm{H}}_2\mathrm{O}\right)}_n\right]}^{2+}\to {\left[\mathrm{Ca}\mathrm{OH}{\left({\mathrm{H}}_2\mathrm{O}\right)}_{n-k}\right]}^{+}+{\mathrm{H}}_3{\mathrm{O}}^{+}{\left({\mathrm{H}}_2\mathrm{O}\right)}_{k-2} $$

Similarly, Armentrout and co-workers have measured the charge-separation barriers for reaction (1) for *n* = 2 and 3 and determined water molecule–binding energies using threshold collision-induced dissociation [11, 12]. Williams and co-workers used blackbody radiation to measure binding energies and infrared laser spectroscopy to probe more extensive Ca^2+^ hydration [13–15]. Binding energies for Ca^2+^ complexes containing both water molecules and methanol molecules have been determined by Bruzzi and Stace [16, 17]. Theory has provided support for the conditions necessary to promote reaction (1) as a function of the number of water molecules present in these doubly charged complexes [18, 19].

With the exception of methanol as a ligand [18–20], there appear to have been no other experimental studies of the gas phase coordination and chemistry of the calcium dication in association with high members of the alcohol series. Likewise, liquid-phase studies in the form of experiments and computer simulations have focused primarily on the Ca^2+^/methanol system and the identification of solvation shells [21, 22]; however, Palka and Hawlicka did include longer-chain alcohols in their calculations on the competition between water and alcohol molecules for the solvation of Ca^2+^ [23]. Connections between the results presented here and gas-phase reactions observed between singly charged alkaline earth metals and ROH molecules will be discussed below.

## Experimental Section

The apparatus used for generation, identification, and detection of gas-phase multiply charged metal–ligand complexes has been described extensively in previous publications [24, 25]. Briefly, mixed neutral clusters were produced by the adiabatic expansion of a gas mixture consisting of alcohol molecules entrained with argon through a pulsed supersonic nozzle. This mixture was generated by flowing argon through a reservoir holding a sample of the alcohol and adjusting the temperature of the latter with an ice bath to achieve optimum signal intensity. Neutral clusters of varying composition, including Ar_*m*_, Ar_*m*_(ROH)_*n*_, and (ROH)_*n*_, then passed through a region where calcium vapor (~ 10^−2^ mbar) was generated by a Knudsen effusion cell (DCA Instruments, EC-40-63-21) operating at 590 °C. Neutral calcium atoms colliding with the cluster beam produced mixed neutral clusters thought to be of the form CaAr_*m*_(ROH)_*n*_. Since the beam consisted of a wide range of mixed clusters, a shutter at the exit to the oven was used to confirm the presence of calcium by noting differences in signal intensity with the shutter open and closed.

Neutral clusters, some of which contained a single metal atom, then entered the ion source of a high-resolution reverse geometry double focusing mass spectrometer (VG-ZAB-E), where they were subjected to electron ionization at energies of between 70 and 100 eV. Since only ions rather than neutral complexes were detected, it is very likely that extensive ligand evaporation took place following ionization, which is believed to help reduce the internal energy content of complexes. Accordingly, under most experimental conditions, no ion complexes of the form [Ca(Ar)_*m*_(ROH)_*n*_]^z+^ were detected. Instead, the recorded mass spectra contained a wide range of both singly and doubly charged complexes formed from different combinations of calcium atoms with each of the alcohol molecules. Also present were very intense signals arising from (ROH)_*n*_H^+^ ions and their fragmentation products. Fortunately, the resolution of the mass spectrometer was sufficient to allow a clear separation of the signals of interest.

In order to monitor the fragmentation patterns of [Ca(ROH)_*n*_]^2+^ complexes, extensive use has been made of the MIKES (mass-analyzed ion kinetic energy spectroscopy) technique [26]. Ions with a specific *m/z* value were selected using a magnet, and the products of their decay were then identified as they passed through an electrostatic analyzer (ESA). These measurements can provide evidence of the fragmentation pathways adopted by an ion of a certain size, which in turn can often be used to infer details of composition and structure. The MIKES technique can be used to study both unimolecular (metastable) decay and processes induced by collisional activation. A collision cell located between the magnet and the ESA allowed ions to enter and exit, while differential pumping located close to the cell ensured that the background pressure in the flight tube as a whole did not change significantly. Gas (nitrogen) was introduced into the cell via a needle valve, and a sufficient amount was added to reduce the intensity of the precursor ion by ~ 50%. In all cases, fragmentation was being detected in the 2nd field-free region (2nd *ffr*) between the magnetic and electric sectors.

Under normal circumstances, the laboratory-frame kinetic energy of a fragment ion, *E*_f_, can be calculated from the following expression [26]:

2 $$ {E}_{\mathrm{f}}=\frac{m_{\mathrm{f}}{q}_{\mathrm{p}}}{m_{\mathrm{p}}{q}_{\mathrm{f}}}{E}_{\mathrm{p}} $$

where *m*_f_ and *m*_p_ are the masses of the fragment and precursor ions, respectively, *q*_f_ and *q*_p_ are their charges, and *E*_p_ is the initial kinetic energy of the precursor ion. By setting the ion source voltage to 5 kV, the electrostatic analyzer can be scanned to transmit ions with laboratory-frame kinetic energies from 10 keV downwards. Those ions that are recorded between 5 and 10 keV can only have come from reactions involving electron loss by the precursor ion. In order to accurately differentiate between processes taking place within the collision cell and those occurring elsewhere within the flight tube, a voltage, *V*_c_, can be applied to the cell to give a separate, collision-induced signal that can be detected at a kinetic energy *E*_fc_ given by the expression

3 $$ {E}_{\mathrm{f}\mathrm{c}}=\frac{m_{\mathrm{f}}{q}_{\mathrm{p}}}{m_{\mathrm{p}}{q}_{\mathrm{f}}}{E}_{\mathrm{p}}+\frac{m_{\mathrm{p}}{q}_{\mathrm{f}}-{m}_{\mathrm{f}}{q}_{\mathrm{p}}}{m_{\mathrm{p}}{q}_{\mathrm{f}}}{V}_{\mathrm{c}} $$

The precise value of the voltage applied to the cell was calibrated using the known collision-induced reactions of CO_2_^2+^.

The MIKES technique has been employed to study two types of unimolecular (metastable) fragmentation that takes place in the absence of collisions and where fragmentation relies on the presence of residual internal energy remaining after electron ionization. As will be seen, following ionization sufficient internal energy remains in the complexes to promote two separate pathways: (i) unimolecular charge separation or Coulomb fission (UCS) in small complexes to give products of the form CaOH^+^, ROH_2_^+^, and R^+^ and (ii) neutral molecule loss in larger complexes. In contrast, collisional activation will be shown to promote a wide range of processes, including electron capture–induced dissociation. In all of the examples that follow, it is the heavier, metal-containing fragment ion that is detected; attempts to detected the lighter R^+^ or ROH_2_^+^ fragments have not been successful because of the much higher levels of instrumental discrimination and background interference.

## Results and Discussion

Similar to earlier studies of [Mg(ROH)_*n*_]^2+^ complexes [6, 27, 28], the equivalent Ca^2+^ system exhibits a series of generic reactions involving both UCS and those induced by the presence of a collision gas, which in this case was nitrogen. The latter reactions can be summarized as follows:

4 $$ {\left[\mathrm{Ca}{\left(\mathrm{ROH}\right)}_n\right]}^{2+}+{\mathrm{N}}_2\to {\left[\mathrm{Ca}{\left(\mathrm{ROH}\right)}_{n-k}\right]}^{2+}+k\ \mathrm{ROH}+{\mathrm{N}}_2 $$

5 $$ {\left[\mathrm{Ca}{\left(\mathrm{ROH}\right)}_{\mathrm{n}}\right]}^{2+}+{\mathrm{N}}_2\to {\mathrm{Ca}}^{+}{\left(\mathrm{ROH}\right)}_{n-k}+k\ \mathrm{ROH}+{{\mathrm{N}}_2}^{+} $$

6 $$ {\left[\mathrm{Ca}{\left(\mathrm{ROH}\right)}_n\right]}^{2+}+{\mathrm{N}}_2\to {\mathrm{CaOH}}^{+}{\left(\mathrm{ROH}\right)}_{n-k-\mathrm{l}}+\mathrm{R}+k\ \mathrm{ROH}+{{\mathrm{N}}_2}^{+} $$

7 $$ {\left[\mathrm{Ca}{\left(\mathrm{ROH}\right)}_n\right]}^{2+}+{\mathrm{N}}_2\to {\mathrm{CaOR}}^{+}{\left(\mathrm{ROH}\right)}_{n-k-1}+k\ \mathrm{ROH}+\mathrm{H}+{{\mathrm{N}}_2}^{+} $$

Steps (5)–(7) are identified as electron capture–induced dissociation (ECID) as opposed to intracluster charge transfer; the occurrence of ECID is consistent with a previous interpretation of data on [Mg(ROH)_*n*_]^2+^ and [Mg(NH_3_)_*n*_]^2+^ complexes [6, 29]. In each case, the reason for this conclusion is that the kinetic energy profiles that accompany these steps are all comparatively narrow, which is in contrast to those reactions that can be unambiguously identified as involving intracluster charge transfer. The latter process results in Coulomb repulsion between the reaction products as they separate, and this leads to much broader kinetic energy profiles. The earlier study of [Mg(NH_3_)_*n*_]^2+^ complexes [29] illustrated the important contribution electron capture makes to the collision-induced fragmentation of doubly charged, metal-containing clusters. This work also illustrated how different collision gases influenced the efficiency of electron capture, and at the laboratory-frame kinetic energies used in these experiments, it has been found that charge reduction processes have comparatively high cross sections (~ 20 Å^2^) [30]. At the center of mass velocities used in these experiments, cross sections of this magnitude favor forward scattering, which helps to explain why the nature of the reaction products, i.e., steps (5)–(7), is quite different from that observed when dication complexes are subjected to comparatively low-energy collisions [11, 12]. A study of neutral molecule loss from hydrogen cluster ions at high collision energies also recorded collision cross sections of the order of ~ 20 Å^2^ [26], which would account for the presence of reaction (4) in many of the MIKE spectra presented below. Individual examples of UCS will be discussed as when they appear in the MIKE spectra.

As a function of size, *n*, the significance of each of steps (4)–(7) varies according to the nature of the alcohol, ROH, and individual examples will be discussed below. Unlike the equivalent Mg^2+^ system [6], none of the [Ca(ROH)_*n*_]^2+^ complexes appear to undergo the non-dissociative electron capture step:

8 $$ {\left[\mathrm{Ca}{\left(\mathrm{ROH}\right)}_n\right]}^{2+}+{\mathrm{N}}_2\to {\mathrm{Ca}}^{+}{\left(\mathrm{ROH}\right)}_n+{{\mathrm{N}}_2}^{+} $$

This absence could be as a consequence of the mismatch between the ionization energy of N_2_ (15.63 eV) and the electron affinity of Ca^2+^ (11.88 eV). In contrast, the close match between N_2_ and Mg^2+^ (15.09 eV) could favor electron transfer without dissociation. Since the nitrogen that is introduced as the collision gas has a thermal kinetic energy, no signal for N_2_^+^ is ever observed in these experiments.

Although the technique used to form complexes made it possible to generate dications with values of *n* extending from 2 to 20, those subject to detailed analysis were normally in the range 2 to 6. As will be seen below, these latter values of *n* were sufficient to establish the changes in behavior that were observed as a function of the size and composition for each [Ca(ROH)_*n*_]^2+^ system. Very rarely was *n* = 1 observed, which is attributed to instability due to Coulomb fission.

### Ca^2+^/Methanol Complexes

**Figure 1:**
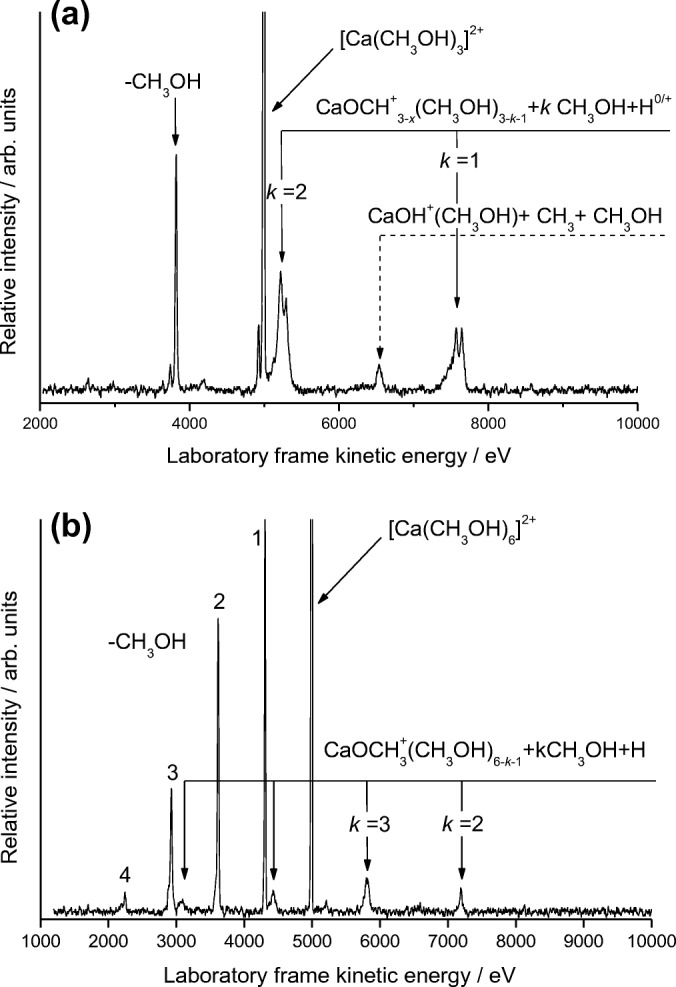
(**a**) MIKE spectrum recorded following the collisional activation of [Ca(CH_3_OH)_3_]^2+^. The notation (0/+) identifies the fact that the *k* = 1 peak includes an underlying USC product C_2_H_5_^+^ that also carries a positive charge. Most of the fragmentation arises from electron capture–induced dissociation where N_2_^+^ is also formed and accompanied by processes that lead to neutral products together with the loss of 3 or 4 H atoms. (**b**) Spectrum recorded following the collisional activation of [Ca(CH_3_OH)_6_]^2+^. The peaks labeled 1–4 correspond to the loss of neutral molecules

A plot of recorded relative intensities of [Ca(CH_3_OH)_*n*_]^2+^ complexes as a function of size *n* is shown in Online Resource 1, where the profile exhibits a very similar trend to those recorded for numerous other metal dication complexes [4, 5, 24, 25] using the pick-up technique. The peak in intensity seen at *n* = 4 does not necessarily imply any favored coordination number, but is more likely to arise as a consequence of the fragmentation patterns of larger complexes, combined with the observation that, in comparison to complexes where *n* > 4, [Ca(CH_3_OH)_4_]^2+^ has a higher binding energy [17]. Since the system is not at equilibrium, the natural tendency is for complexes to fragment downwards towards the most stable unit, which itself often represents a compromise between a high binding energy and unimolecular charge separation driven by excess internal energy. Although *n* = 4 is most frequently the most intense ion in many of the systems studied, that is not always the case [31]. An earlier study of Coulomb fission in a wide range of small metal dication–ligand complexes [20] reported that [Ca(CH_3_OH)_3_]^2+^ showed evidence for the loss of CH_3_OH_2_^+^, but that no similar process could be identified for [Ca(CH_3_OH)_2_]^2+^, which instead loses CH_3_^+^. Very similar observations were made for Mg^2+^ [6]. Figure 1a shows a MIKE scan recorded following the collisional activation of [Ca(CH_3_OH)_3_]^2+^. The presence can be seen of both reactions (6) and (7) above, with the narrow peak profiles, particularly for reaction (6) being indicative of ECID. In contrast to this mix of reaction products, Figure 1b shows the results from a MIKE scan of [Ca(CH_3_OH)_6_]^2+^, where reaction (6) is no longer observed and fragmentation is now dominated by neutral ligand loss together with contributions from reaction (7). This transition from a mixture of CaOH^+^ and CaOCH_3_^+^ fragments to product ions that just contain the latter is gradual, but complete by *n* = 5.

Similar switching reactions involving singly charged alkaline earth metal ions in association with both water and methanol molecules have been the subject of experimental and theoretical studies [32–39]. Since the outcome of electron capture in the experiments discussed here is the generation of a singly charged complex, comparable processes are to be expected. In their study of alkaline-earth metal ions complexed with water, Fuke and co-workers recorded mass spectra showing the presence of two types of ion at low masses: M^+^(H_2_O)_*n*_ and MOH^+^(H_2_O)_*n*_ (M^+^ = Mg or Ca) with the latter becoming the sole cation beyond *n* = 5 [32]. This transition was attributed to a difference in hydration energy, with MOH^+^ being more stable in the presence of large numbers of water molecules [32]. For the case of complexes between singly charged alkaline earth metals and methanol, both photo- and collisional-activation studies have shown evidence of switching reactions [35–39]. As part of their study of doubly charged metal ions complexed with solvent molecules, Kohler and Leary studied the low-energy collision-induced dissociation of [Ca(CH_3_OH)_4_]^2+^ [18, 19], where they observed CaOCH_3_^+^ as the dominant fragment, accompanied by a much weaker signal for CaOH^+^. Direct, collision-induced bond cleavage was assumed to be responsible for the appearance of these fragments, which, given the comparatively low collision energies used by Kohler and Leary [18, 19], is probably going to be more efficient than ECID. A theoretical analysis by Chan et al. [34] of the intracluster reaction channels available to [Ca(CH_3_OH)_*n*_]^2+^ and [Mg(CH_3_OH)_*n*_]^2+^ complexes concluded that switching arises from a more rapid decline in the reaction barrier to the elimination of H from the OH group than for the elimination of CH_3_. As *n* increases, solvation helps to stabilize the more polarizable CaOCH_3_^+^ ion core [34].

### Ca^2+^/Ethanol Complexes

**Figure 2:**
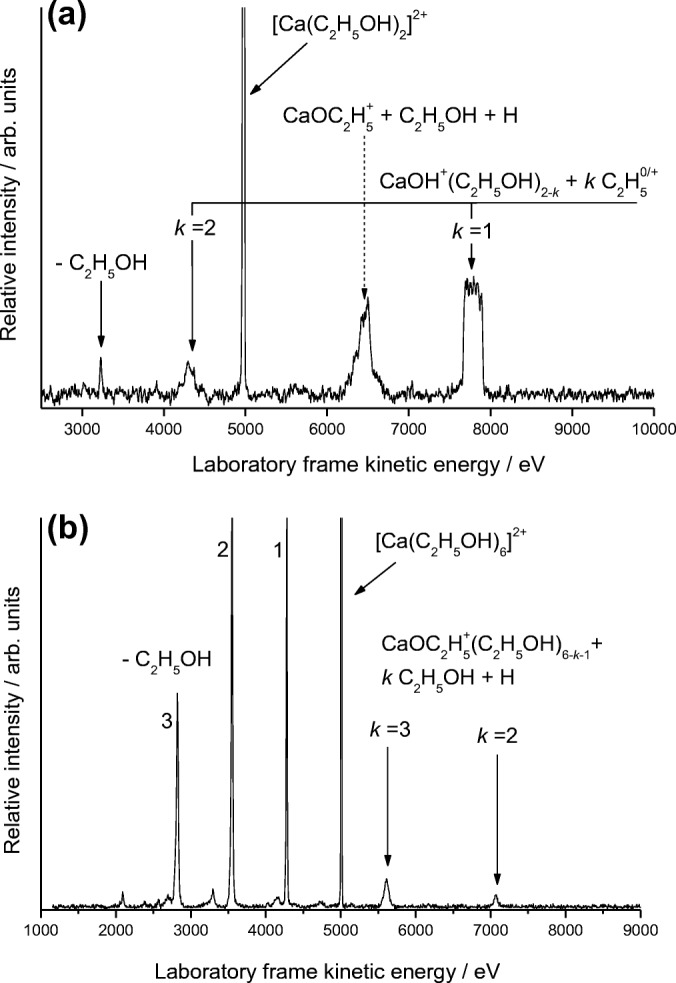
(**a**) As for Fig. 1a, but following the collisional activation of [Ca(C_2_H_5_OH)_2_]^2+^. In this case, the UCS product is C_2_H_5_^+^. (**b**) Spectrum recorded following the collisional activation of [Ca(C_2_H_5_OH)_6_]^2+^. The peaks labeled 1–3 correspond to the loss of neutral molecules

**Figure 3:**
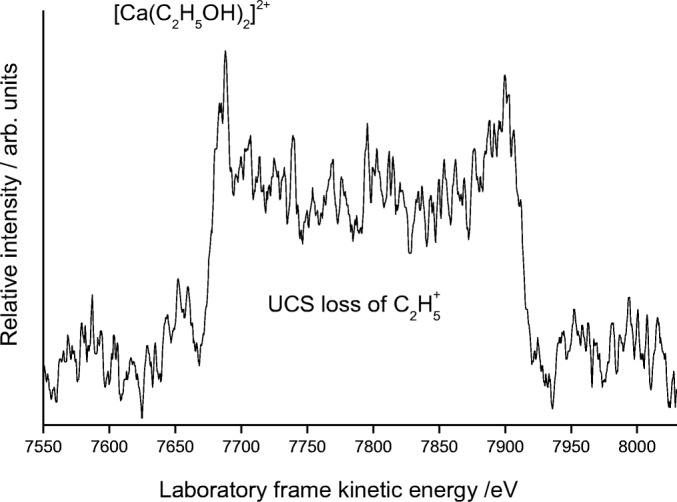
Kinetic energy profile from an expanded section of Fig. 2a, but recorded in the absence of any collision gas. The peak arises from the unimolecular charge separation step: [Ca(C_2_H_5_OH)_2_]^2+^ → Ca^+^OH(C_2_H_5_OH) + C_2_H_5_^+^

**Figure 4:**
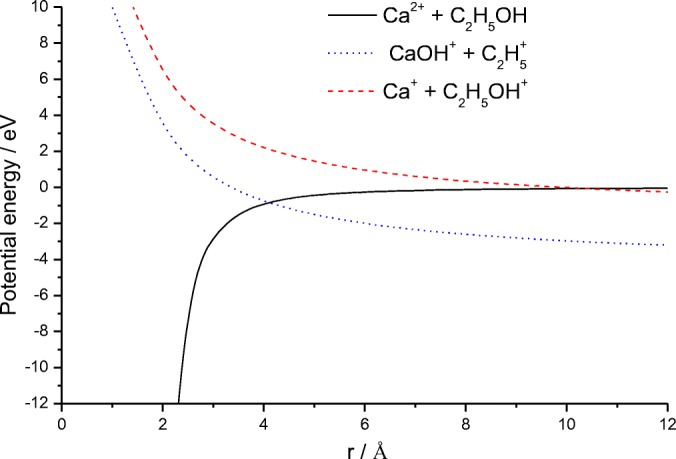
Calculated potential energy curves for Ca^+^ + C_2_H_5_OH, showing the attractive ion-dipole and ion-induced dipole curves and the repulsive coulomb curve corresponding to the appearance of different charge separation products. The curve leading to CaOC_2_H_5_^+^ + H^+^ has been omitted because of the high energy required to generate H^+^

Figure 2a shows fragment ions recorded following collisions between N_2_ and [Ca(C_2_H_5_OH)_2_]^2+^, where two separate series of fragment ions is observed and centered on the formation of either CaOC_2_H_5_^+^ or CaOH^+^. The strong peak at ~ 7800 eV includes several processes contributing to the formation of CaOH^+^(C_2_H_5_OH), and removal of the collision gas reveals the metastable peak shown in Figure 3. The dish shape to the peak is indicative of Coulomb fission and corresponds to unimolecular charge separation to give C_2_H_5_^+^ as the complementary fragment ion. Earlier analysis of similar charge transfer processes made use of simple one-dimensional potential energy curves to account for the charged fragments [6, 25]. A series of curves depicting possible outcomes for Ca^2+^ + C_2_H_5_OH is shown in Figure 4, where the various positions have been determined through a combination of reaction exothermicities and ion-dipole and ion-induced dipole interaction energies. At best, the curves are semi-quantitative since there is no curve corresponding to proton transfer, as this would require two molecules, and no account has been taken of the influence additional molecules might have on stabilizing either the precursor or fragment ions. Tonkyn and Weisshaar [40] have previously used curves of the type shown in Figure 4 to differentiate between H^−^ transfer and electron transfer in collisions between metal dications and molecules; however, in the present case, the outcome is going to be different because the complexes are starting from within the potential well. As Figure 4 shows, the first curve crossing to be encountered correlates with Ca^+^OH and C_2_H_5_^+^ as reaction products. Slightly higher in energy is a curve crossing that correlates with the products Ca^+^ + C_2_H_5_OH^+^. Since metastable decay is normally associated with the pathway with the lowest energy barrier, the appearance of a peak for the loss of C_2_H_5_^+^ confirms the ordering of the potential curves seen in Figure 4. Applications of a more rigorous theory to the problem comes from the work of El-Nahas et al. [41] who analyzed the structure and stability of the [MgCH_3_OH]^2+^ complex. They concluded that the lowest-energy pathway was that leading to the formation of MgOH^+^ and CH_3_^+^ as charge transfer fragments [41].

By applying a voltage to the collision cell (calibrated to be + 963 V), it is possible to separate reaction steps due to metastable decay from those initiated by collisional activation. Online Resource 1 shows an example where such an approach has been applied to the peak appearing at 7800 eV in Figure 2a. As can be seen, the metastable peak (a) identified in Figure 2a remains in position because it corresponds to decay throughout the entire 2nd *ffr*. However, a second feature appearing at ~ 7500 eV (b) corresponds to processes taking place within the collision cell, which together with CaOH^+^(C_2_H_5_OH) includes a range of singly charged products associated with the loss of one or more H atoms as a result of electron transfer with the nitrogen collision gas. Individual H atom loss could not be resolved in the present experiments, but is evident in previous studies of a similar nature [6, 28], and the peak width probably corresponds to the loss of 3 to 4 H atoms. A MIKE scan of [Ca(C_2_H_5_OH)_3_]^2+^ shows very similar reaction products to those seen for [Ca(C_2_H_5_OH)_2_]^2+^ and includes a strong signal arising from the metastable loss of C_2_H_5_^+^ from the precursor ion. Figure 2b shows the outcome of a scan on [Ca(C_2_H_5_OH)_6_]^2+^ following collisional activation, where it can be seen that, similar to the equivalent methanol complex, the reaction products are now entirely due to the formation of CaOC_2_H_5_^+^(C_2_H_5_OH)_*m*_, with no evidence of fragments containing CaOH^+^. Figure 2b also shows evidence of extensive loss of neutral molecules.

### Ca^2+^/1-Propanol Complexes

**Figure 5:**
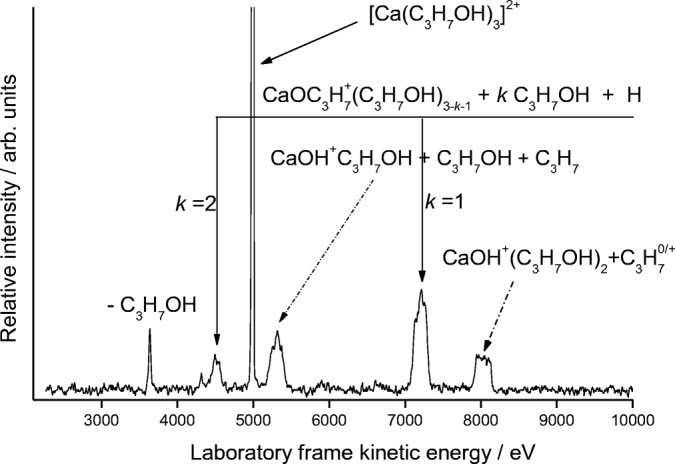
As for Fig. 1a, but following the collisional activation of a complex with 1-propanol, [Ca(C_3_H_7_OH)_3_]^2+^. In this example, the UCS product is C_3_H_7_^+^

Figure 5 shows a MIKE scan recorded following the collision of [Ca(C_3_H_7_OH)_3_]^2+^ ions with nitrogen. Once again, there is a mix of fragment ions corresponding to the formation of both CaOH^+^ and CaOC_3_H_7_^+^, with the former product ion no longer appearing once the precursor contains 6 1-propanol molecules. Both the *n* = 2 and *n* = 3 complexes exhibit strong metastable signals corresponding to the loss of C_3_H_7_^+^, and similar to that shown in Online Resource 1, metastable and ECID signals can be identified separately following the application of a voltage to the peak seen at ~ 8000 eV in Figure 5. An earlier study of strontium/1-propanol complexes, [Sr(C_3_H_7_OH)_*n*_]^2+^, also noted the presence of a strong metastable signal for the appearance of SrOH^+^ when *n* = 2, but failed to appreciate the role played by electron transfer in processes induced by collisional activation in complexes where *n* ≥ 3 [42].

.

### Ca^2+^/2-Propanol Complexes

**Figure 6:**
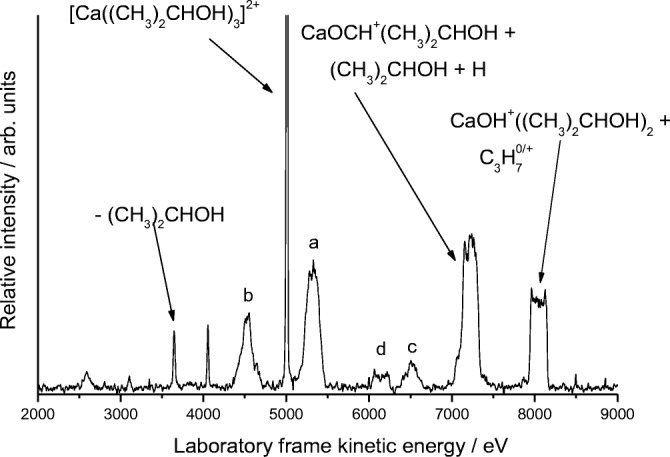
As for Fig. 1a, but following the collisional activation of a complex with 2-propanol, [Ca((CH_3_)_2_CHOH)_3_]^2+^. In this example, the UCS product is C_3_H_7_^+^

As Figure 6 shows, there is a very close match between the MIKE scan recorded for 1-propanol and that shown in Figure 5 for 2-propanol. The peaks labeled a and b denote equivalent steps seen in both systems, whereas the peaks labeled c and d, along with that seen at ~ 4000 eV, cannot be assigned. Peak c is at a position that is equivalent to the loss of (CH_3_)_2_CHOH + 17 amu, and although the loss of H_2_O has been observed in protonated clusters of pure 2-propanol [43], the mismatch in mass is sufficient as to rule out that process here. The alternative is the loss of OH rather than water to give CaCH(CH_3_)_2_^+^HOCH(CH_3_)_2_; however, the formation of a metal–carbon bond has not been reported in other related studies. As with previous examples, the application of a voltage to the collision cell shows that the peak at ~ 8000 eV can be resolved into a strong metastable component for the loss of C_3_H_7_^+^ together with a range of ECID processes centered on the loss of neutral C_3_H_7_ possibly accompanied by the loss of one or two hydrogen atoms.

### Ca^2+^/1-Butanol Complexes

**Figure 7:**
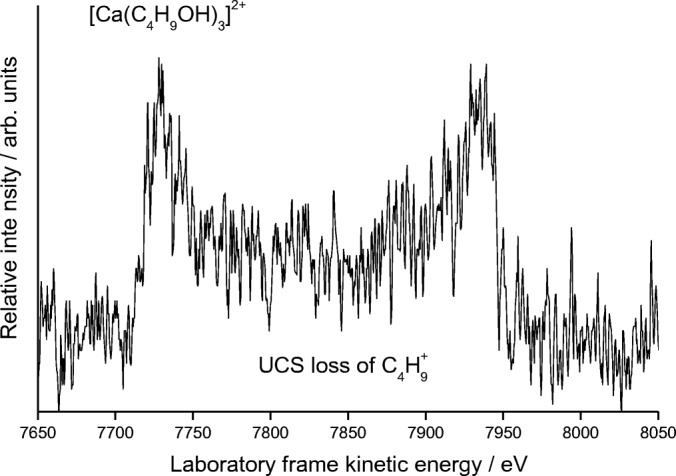
Kinetic energy profile from the UCS decay of a complex with 1-butanol, [Ca(C_4_H_9_OH)_3_]^2+^ recorded in the absence of any collision gas. The peak arises from the unimolecular charge separation step: [Ca(C_4_H_9_OH)_3_]^2+^ → Ca^+^OH(C_4_H_9_OH)_2_ + C_4_H_9_^+^

The collisional activation of individual [Ca(C_4_H_9_OH)_*n*_]^2+^ complexes for small values of *n* resulted in sequences of product ions similar to those seen in previous examples, i.e., based on Ca^+^OC_4_H_9_ and CaOH^+^. Two of the complexes, *n* = 2 and 3, exhibited strong metastable signals, and an example recorded for [Ca(C_4_H_9_OH)_3_]^2+^ is shown in Figure 7. However, this particular complex also undergoes a second unimolecular step in the form of

9 $$ {\left[\mathrm{Ca}{\left({\mathrm{C}}_4{\mathrm{H}}_9\mathrm{OH}\right)}_3\right]}^{2+}\to {\mathrm{C}\mathrm{aOC}}_4{{\mathrm{H}}_9}^{+}\left({\mathrm{C}}_4{\mathrm{H}}_9\mathrm{OH}\right)+{\mathrm{C}}_4{\mathrm{H}}_9{{\mathrm{OH}}_2}^{+} $$

A similar observation was made on the corresponding Mg^2+^ complex, but in that case the two unimolecular reactions were observed for *n* = 4 rather than 3. The collisional activation of [Ca(C_4_H_9_OH)_*n*_]^2+^ complexes for *n* ≥ 4 resulted in product ions centered on the formation of CaOC_4_H_9_^+^, and no further metastable processes were observed.

### Ca^2+^/2-Butanol Complexes

Complexes containing 2-butanol were not the subject of the earlier study involving Mg^2+^ [6]. The fragmentation patterns recorded for these complexes were almost identical to those seen for the equivalent series involving 1-butanol including the two metastable steps equivalent to those shown in Figure 7 and by reaction (9).

### Ca^2+^/*t*-Butanol Complexes

The low vapor pressure of *t*-butanol resulted in very weak signals for this series of complexes. Signal intensity for the precursor ions peaked at *n* = 4 and the fragmentation pattern for this ion showed the presence of products containing CaOC(CH_3_)_3_^+^ and CaOH^+^. However, no metastable processes could be identified and once *n* = 6 almost all ECID signals had disappeared.

### Ca^2+^/Chloroethanol Complexes

**Figure 8:**
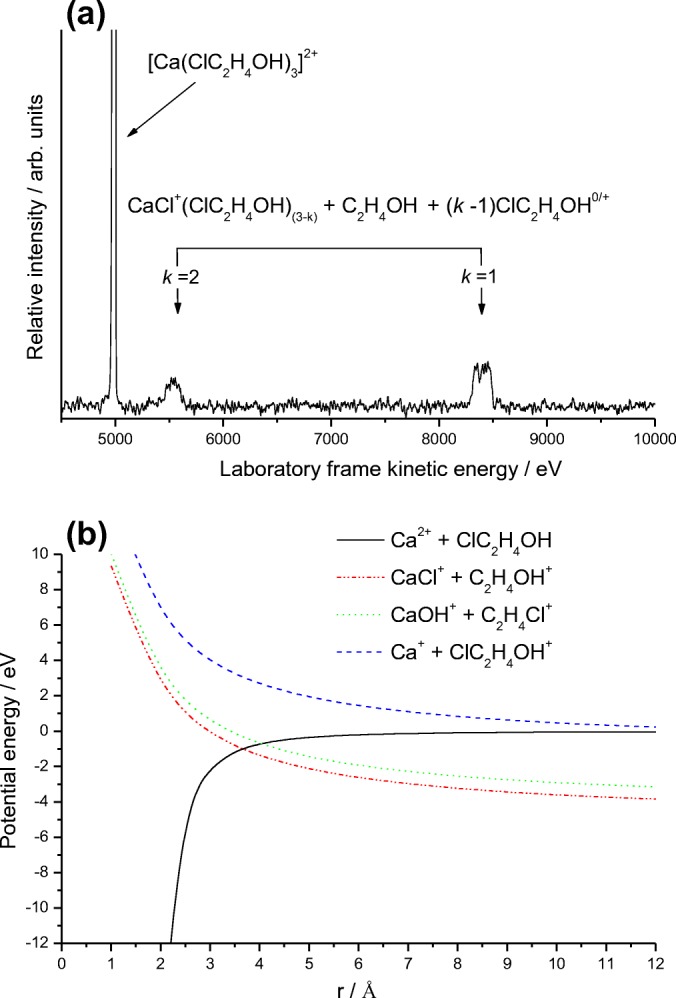
(**a**) As for Fig. 1a, but following the collisional activation of a complex with 1-chloroethanol, [Ca(ClC_2_H_4_OH)_3_]^2+^. Note the complete absence of any of the ECID products identified following similar experiments on complexes with non-substituted alcohol molecules. (**b**) Calculated potential energy curves for Ca^+^ + ClC_2_H_4_OH, showing the attractive ion-dipole and ion-induced dipole curves and the repulsive coulomb curve corresponding to the appearance of different charge separation products

This final example has been chosen in order to explore the preference Ca^+^ might have for extracting a chlorine atom as opposed to the other two dominant products seen for the unsubstituted alcohol molecules studied above. These experiments were run using the more intense Cl^35^ isotope. As can be seen in Figure 8a, the collision-induced fragmentation pattern shows just a single reaction product in the form of fragment ions that are centered on the formation of CaCl^+^. Removal of the collision gas shows the peak seen at ~ 8400 eV to include a strong metastable component arising from the reaction:

10 $$ {\left[\mathrm{Ca}{\left({\mathrm{C}\mathrm{lC}}_2{\mathrm{H}}_4\mathrm{OH}\right)}_3\right]}^{2+}\to {\mathrm{C}\mathrm{aCl}}^{+}{\left({\mathrm{C}\mathrm{lC}}_2{\mathrm{H}}_4\mathrm{OH}\right)}_2+{\mathrm{C}}_2{\mathrm{H}}_4{\mathrm{OH}}^{+} $$

Confirmation that reaction (10) is the most facile charge separation route can be seen in Figure 8b, where an analysis of reaction pathways using simple one-dimension potential energy curves, has been extended to include a route leading to the formation of Ca^+^Cl. As can be seen, the above pathway is the first to be encountered as the reactants move out from the potential well. Since Ca–Cl^+^ and Ca–OH^+^ have comparable bond strengths, 435 and 433 kJ mol^−1^, respectively [44], what appears to favor formation of the former over that of the latter is the ~ 55 kJ mol^−1^ difference in bond energy between Cl–C_2_H_5_OH and HO–C_2_H_5_Cl, which are estimated to be ~ 335 kJ mol^−1^ and ~ 390 kJ mol^−1^, respectively [44].

## Conclusion

An extensive series of experiments have been undertaken to examine the influence different aliphatic alcohols have on the behavior of the dication complexes [Ca(ROH)_*n*_]^2+^. The results provide evidence of two types of switching reactions, each being associated with separate fragmentation pathways and each having a clearly recognizable kinetic energy release profile. The first switching reaction is induced by electron capture from a collision gas (nitrogen) and proceeds via the generation of a singly charged Ca^+^(ROH)_*n*_ species. This ion subsequently decays to give a charged core which takes the form of either CaOH^+^ or CaOR^+^, with the latter gaining in intensity as *n* increases. This pattern of behavior matches that seen for a wide range of singly charged alkaline earth/ROH complexes, including those where R=H [32–39]. A second form of switching reaction is summarized in Table 1 and is associated with those doubly charged [Ca(ROH)_*n*_]^2+^ ions that undergo unimolecular charge separation within the limited values of *n* as shown. These reactions give in each case two singly charged fragments either of the form CaOH^+^ as a core together with R^+^ or CaOR^+^ as a core together with ROH_2_^+^. This latter behavior could be attributed to complexes being formed where at least one molecule occupies a hydrogen-bonded site in the second solvation shell. The geometry of this arrangement would then facilitate the removal of a proton from a first-shell molecule to leave CaOR^+^ as a core. Examples of where a second solvation shell begins to develop before completion of the first shell have been identified previously in complexes with Mg^2+^ but not in any involving Ca^2+^ [6, 13, 14, 45]. Also included in Table 1 are equivalent results recorded for Mg^2+^ complexes. A qualitative trend observed for the latter was that for the generic metastable step:

**Table 1:** Summary of the Metastable, Unimolecular, Charge Separation Reactions (UCS) Observed When Each of the Alcohol Molecules Is Coordinated to Ca^2+^

Ligand	UCS(Ca^2+^)	*n*(Ca^2+^)	*n*(Mg^2+^)^$^	Charge separation	Proton transfer	IE (R)^#^
CH_3_OH^!^	Yes	3	3	CH_3_^+^ (*n* = 2)	CH_3_OH_2_^+^ (*n* = 3)	9.84
C_2_H_5_OH	Yes	3	3	C_2_H_5_^+^ (*n* = 2,3)	–	8.12
1-C_3_H_7_OH^%^	Yes	3	4	C_3_H_7_^+^ (*n* = 2,3)	–	8.09
2-C_3_H_7_OH	Yes	3	–	C_3_H_7_^+^ (*n =* 2,3)	–	7.37
1-C_4_H_9_OH	Yes	3	4	C_4_H_9_^+^ (*n* = 2,3)	C_4_H_9_OH_2_^+^ (*n* = 3)	8.02
2-C_4_H_9_OH	Yes	3	–	C_4_H_9_^+^ (*n* = 3)	C_4_H_9_OH_2_^+^ (*n* = 3)	7.25
*t*-C_4_H_9_OH	No	–	4	–	–	6.80
ClC_2_H_4_OH	Yes	–	–	C_2_H_4_OH^+^	–	6.70

Also given as IE(R) are the ionization energies of the radicals that are produced as complementary ions during charge separation. For [M(ROH)_*n*_]^2+^ complexes, *n* is the maximum values for which UCS is observed, where M is either Ca or Mg

^!^Taken from ref. [20]

^$^Data for Mg^2+^ complexes. Taken from ref. [6]

^#^Ionization energy in eV (taken from the NIST Chemistry WebBook)

^%^The complex [Sr(1-C_3_H_7_OH)_2_]^2+^ was also observed to undergo this UCS step [42]

11 $$ {\left[\mathrm{Mg}{\left(\mathrm{ROH}\right)}_n\right]}^{2+}\to {\mathrm{MgOH}}^{+}{\left(\mathrm{ROH}\right)}_{n-1}+{\mathrm{R}}^{+} $$

The value of *n* required to stabilize each complex against unimolecular charge separation increased as the ionization energy of R declined; the relevant data are given in Table 1. There appears to be no evidence of a similar trend in the data recorded for Ca^2+^, in that all of the complexes require four alcohol molecules to stabilize against charge separation. However, there are differences between Mg^2+^ and Ca^2+^, in that the higher ionization energy of the former requires five rather than four molecules of both 1-propanol and 1-butanol to stabilize against UCS.

## Electronic supplementary material

ESM 1 (PDF 212 kb)
