# Supplementary material for: The Solvation of Ca2+ with Gas Phase Clusters of Alcohol Molecules
Source: J Am Soc Mass Spectrom. 2019 Jul 8;30(9):1768–78. doi: 10.1007/s13361-019-02263-x (PMC6695372; doi:10.1007/s13361-019-02263-x)

Online Resource 1

The solvation of Ca^{2+} with gas phase clusters of alcohol molecules.

Khadar Duale and Anthony J. Stace*
School of Chemistry, The University of Nottingham, University Park, Nottingham NG7 2RD, United Kingdom.
* Corresponding author: Anthony.stace@nottingham.ac.uk

Figure 1. Plot of the relative intensities of doubly charged $[Ca(CH_3OH)_n]^{2+}$ ions as a function of *n*.

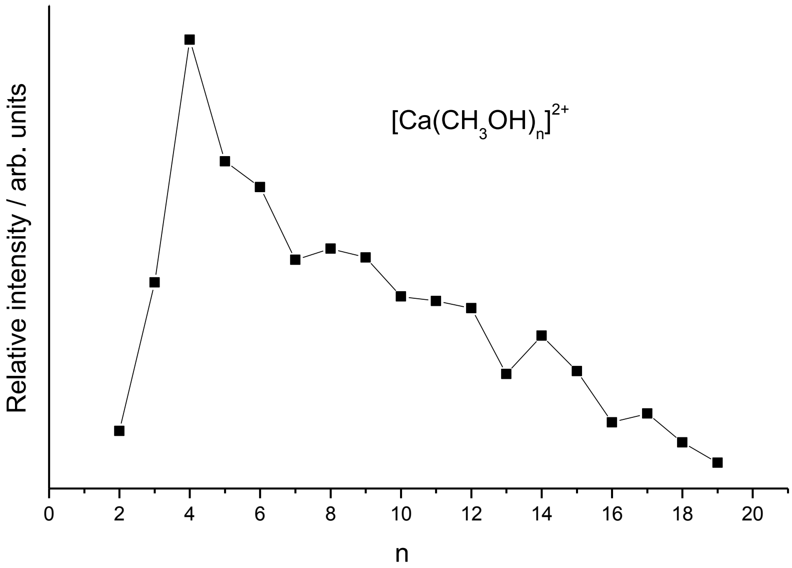

Figure 2. As for Figure 5, but from a MIKE scan of $[Ca(C_2H_5OH)_2]^{2+}$ recorded in the presence of a collision gas and with the collision cell floated at a voltage of +963 V. Peak a is still predominantly formation of $Ca^{+}OH(C_2H_5OH)$ from UCS, and peak b includes a range of singly charged products, including $Ca^{+}OH(C_2H_5OH)$, formed as a consequence of electron capture. Peak a no longer has the pronounced dish-shape shown in figure 3 because gas leakage from the collision cell also promotes ECID processes in the flight tube.

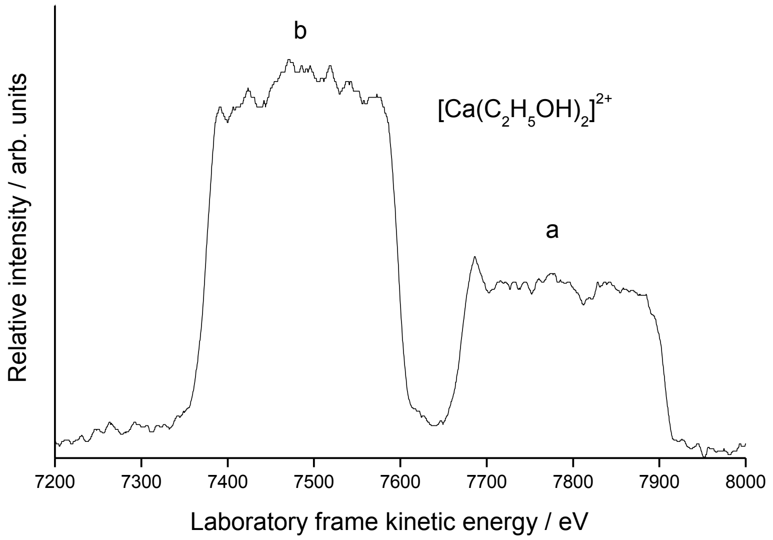

Supplement: Supplementary file 1 — (PDF 212 kb) [file 13361_2019_2263_MOESM1_ESM.pdf]
